# Global developmental delay: comparison of developmental profiles between gene-positive/suspicious positive and gene-negative cases

**DOI:** 10.1038/s41390-025-04085-y

**Published:** 2025-04-30

**Authors:** Ling Shan, Miao-Shui Bai, Han-Yu Dong, Jun-Yan Feng, Tian-Tian Wang, Zakaria Ahmed Mohamed, Chun-Yue Miao, Fei-Yong Jia

**Affiliations:** 1 Department of Developmental and Behavioral Pediatrics, Children’s Medical Center, The First Hospital of Jilin University, Changchun, China; 2 The Child Health Clinical Research Center of Jilin Province, Changchun, China; 3 Jilin University, Changchun, China

## Abstract

**Background:**

Global developmental delay (GDD) is associated with genetic abnormalities; however, the specific clinical and developmental features that should trigger genetic testing remain unclear. In this study, we explored this issue.

**Methods:**

A total of 126 children with GDD were recruited for this study. Comprehensive medical histories and physical examination data were collected for all participants. The Chinese adaptation of the Griffiths Mental Development Scales was used to assess neurodevelopmental outcomes. Genetic variations were analyzed through trio-based whole exome sequencing and proband whole genome sequencing. A comparative analysis of the clinical characteristics was conducted between children with gene-positive/suspicious positive results (i.e., the mutation is deleterious or potentially deleterious, and the inheritance pattern and phenotype are matched) and those with negative results.

**Result:**

The positive/suspicious positive rate of genes was 46.8%. The locomotor, performance, and general quotients were lower in the gene-positive/suspicious positive group than the gene-negative group (*p* < 0.05), and the lower the locomotor ability, the higher the gene positive/suspicious positive rate (*p* < 0.05).

**Conclusion:**

Children with GDD and genetic abnormalities exhibited poorer locomotor, performance, and general developmental quotients compared to those without genetic mutations. Furthermore, individuals with poorer locomotor ability should be prioritized for genetic testing.

**Impact:**

- This study aimed to compare the clinical and developmental profiles of children with GDD who test positive or suspiciously positive for genetic abnormalities with those who test negative, and to identify key clinical features that may serve as indicators for genetic testing.
- It highlights that children with GDD and genetic abnormalities exhibited poorer locomotor, performance, and general developmental quotients compared to those without genetic mutations. Individuals with poorer locomotor ability should be prioritized for genetic testing.
- The findings supplement existing literature by providing insights to guide clinicians on determining which children with GDD should be considered for genetic testing.

## Introduction

Global developmental delay (GDD) is characterized by significant delays in at least two developmental domains before the age of 5 years.^1^ The prevalence of GDD ranges from 1% to 3%.^2^ GDD is a transition type of intellectual disability (ID), as most children diagnosed with ID usually exhibit GDD during the early developmental period, however, not all children with GDD will progress to ID.^3^ GDD/ID represents a complex disorder resulting from the interplay of genetic and environmental factors. Genetic factors, including small insertions and deletions (INDELs), single nucleotide variants (SNVs), copy-number variants (CNVs), and aneuploidies, are implicated in 30%–60% of GDD/ID cases.^4–6^

Recent advances in next-generation sequencing technologies, particularly the widespread adoption of trio-based whole-exome sequencing (trio-WES) and whole-genome sequencing (WGS), have significantly improved the detection of SNVs and CNVs. These technologies have led to the identification of genetic causes in a growing number of cases previously categorized as unexplained or idiopathic GDD/ID.^7,8^ To date, over 2,500 genes associated with the pathogenesis of GDD/ID have been identified, and this number continues to rise annually.^9^ A recent systematic review by the American College of Medical Genetics and Genomics (ACMG) recommended that WES and WGS should be considered as first-or second-tier diagnostic tests for patients presenting with congenital anomalies, developmental delay, or ID.^10^ Despite these advancements, the clinical application of trio- WES and WGS remains limited by high substantial costs.

From a clinical perspective, it is essential to identify the phenotypic characteristics that warrant genetic testing in children with GDD. Furthermore, it is critical to determine whether children with GDD who possess genetic abnormalities exhibit inferior developmental trajectories. Existing genetic research on GDD and ID has primarily focused on identifying novel pathogenic genes, often overlooking these critical clinical questions. Therefore, the objective of this study is to compare the clinical and developmental profiles of children with GDD who test positive or suspiciously positive for genetic abnormalities to those who test negative. The findings from this research will provide valuable insights to guide evidence-based clinical decision-making.

## Methods

### Participants

A total of 126 children aged 18–60 months, predominantly presenting with GDD were recruited from September 2021 to January 2022 at the Outpatient Department of Developmental and Behavioral Pediatrics, the First Hospital of Jilin University, Changchun, China. The study was approved by the ethics committee of the First Hospital of Jilin University, and written informed consent was obtained from the legal guardians of all participants. GDD diagnoses were made according to the criteria outlined in the Diagnostic and Statistical Manual of Mental Disorders, Fifth Edition (DSM-5). Neurodevelopmental symptoms were assessed using the Chinese version of the Griffiths Mental Development Scales (GDS-C). Genetic analysis was conducted via trio-WES and proband WGS. Children with cerebral palsy, ataxia, motor neurodegenerative diseases, intracranial infection, craniocerebral injuries, or intracranial hemorrhage as well as those whose legal guardians did not provide informed consent, were excluded from the study.

### Measurements

#### Neurodevelopment assessment

The GDS-C was used to assess the neurodevelopmental outcomes of the participants. The GDS-C is a reliable and valid developmental assessment tool widely used in China containing the following 5 independent subscales for assessing the developmental level of children aged 0–2 years: locomotor (A scale), personal–social skills (B scale), hearing–speech (C scale), eye–hand coordination (D scale), and performance (E scale).^11^ An additional scale, practical reasoning (F scale), is applied when assessing children aged 3–8 years. Thereafter, developmental quotients (DQs) were calculated for each subscale by dividing the developmental age by the chronological age (DQ = [developmental age/chronologic age] × 100).^12^ The DQs of each scale are known as the AQ, BQ, CQ, DQ, EQ, and FQ, respectively, and the average score of all subscales is considered to be the general quotient (GQ). The GQ and each of the 6 subscale quotients have a mean of 100 points (standard deviation [SD], 15 points). A GQ or subscale quotient of <70 points (>2 SD below the mean) indicates a significant delay in development, whereas a quotient of ≥70 points indicates a mild or absent delay.^13^ In our analysis, subscale quotients were classified as follows: normal or mild defect (DQ ≥ 70), moderate defect (55 ≤ DQ < 70), and severe defect (DQ < 55).

#### Genetic testing

Genetic variants were identified through trio-WES and proband WGS. Initially, trio-WES was performed on the proband and both parents, followed by WGS of the proband to investigate mitochondrial genomic variations, and intron regions, among others.

Genomic DNA was extracted using the Blood Genome Column Medium Extraction Kit (Kangweishiji, China) following the manufacturer’s instructions. The exonic regions and flanking splice junctions of the genomic DNA from the proband and parents were captured by using the xGen Exome Research Panel v2.0 (IDT, Coralville, IA). Finally, the libraries captured were sequenced on a DNBSEQ-T7 series sequencer (MGI Tech Co., Ltd., Shenzhen, China) with the following parameters: PE150 and ≥11.6 million reads. CNV, WGS and WES were performed by the Beijing Chigene Translational Medicine Research Center (Beijing, China).

#### Procedure

The procedure for the evaluation and diagnosis of GDD are described in detail in our previous publication.^14^ Blood samples for WES and WGS were collected from consenting subjects. After obtaining informed consent, 4 mL of peripheral blood was drawn from each child and parent into ethylenediaminetetraacetic acid anticoagulant tubes. Blood collection was carried out by trained nurses following strict sterile and standardized protocols. Samples were immediately stored at 4 °C, delivered to the laboratory within 72 h, and then stored at −80 °C until DNA extraction.

### Statistical methods

Data analysis was conducted using SPSS Statistics version 22.0 (IBM Corp., Armonk, NY). Continuous data are presented as mean ± SD, while categorical data are presented as numbers and percentages. The chi-squared goodness-of-fit test was used for within-group comparisons of different indicator rates, and the chi-squared test or Fisher’s exact test was used for between-group comparisons of different indicator rates. For comparison of the 2 groups, normal distributed data were analyzed using a t-test; nonparametric data were analyzed using a Mann-Whitney U test. We tested for linear trends of the gene-positivity rate across different gross motor ability groups using the chi-squared linear trend test. All tests were 2-sided, with *p* < 0.05 used as the significance threshold.

## Results

### Sociodemographic characteristics of GDD patients

This study included 126 children, with their demographic characteristics are shown in Table 1. The mean age of participants was 37.07 ± 12.90 months (range 18–60 months), and the sample consisted of 83 boys and 43 girls (a ratio of 1.9:1). The preterm birth rate was 4.8%. Among the 126 participants, 7.9% had family history of neuropsychiatric disorders, 5.6% had epilepsy, 5.6% had macrocephaly (>2 SD), and 8.7% had microcephaly (<2 SD).

**Table 1:** Comparison of basic data between gene-positive/suspicious positive group and gene-negative group of GDD^a^.

		GDD (*n* = 126)	Positive/suspicious positive group (*n* = 59)	Negative group (*n* = 67)	*t/u/χ2/Fisher*	*p*
Male/female		83/43 (1.9:1)	34/25 (1.4:1)	49/18 (2.7:1)	3.356	0.067
Age (months)		37.07 ± 12.90	37.63 ± 13.40	36.59 ± 12.52	0.453	0.652
Maternal age at conception (years)		29.00 ± 4.05	29.49 ± 4.09	28.57 ± 3.98	0.739	0.460
Paternal age at conception (years)		30.84 ± 5.02	31.29 ± 5.62	30.45 ± 4.43	0.260	0.795
Preterm birth, *n* (%)		6 (4.8%)	3 (5.1%)	3 (4.5%)	–	1.000
Family history of neuropsychiatric disorders, *n* (%)		10 (7.9%)	4 (6.8%)	6 (9.0%)	–	0.749
	Depression, *n* (%)	4 (3.2%)	1 (1.7%)	3 (4.5%)	–	–
	Cerebral palsy, *n* (%)	1 (0.8%)	1 (1.7%)	0 (0)	–	–
	Schizophrenia, *n* (%)	3 (2.4%)	2 (3.4%)	1 (1.5%)	–	–
	Intellectual disability, *n* (%)	2 (1.6%)	0 (0)	2 (3%)	–	–
Epilepsy, *n* (%)		7 (5.6%)	6 (10.2%)	1 (1.5%)	–	0.034^*^
Abnormal head circumference, *n* (%)		18 (14.3%)	14 (23.7%)	4 (6.0%)	8.080	0.004**
	Macrocephaly (>2 SD), *n* (%)	7 (5.6%)	5 (8.5%)	2 (3.0%)	–	0.251
	Microcephaly (<2 SD), *n* (%)	11 (8.7%)	9 (15.3%)	2 (3.0%)	–	0.015*

*GDD* global developmental delay.

*p < 0.05; **p < 0.01.

^a^Data are mean ± SD or number (%).

### Composition and classification of pathogenic/possibly pathogenic mutations in GDD patients

Of the 126 children with GDD, 59 (46.8%) were identified as gene-positive/suspicious positive, with a total of 68 pathogenic/possibly pathogenic mutations detected. These included 17 (25.0%) CNVs and 51 (75.0%) SNVs/INDELs. Among these mutations, 36 (52.9%) were de novo while 32 (47.1%) were inherited from parents (Supplemental Table S1 and S2).

### Comparison of clinical data and GDS-C scores between gene-positive/suspicious positive and gene-negative children with GDD

There was no significant difference in age, sex ratio, maternal/paternal age at conception, preterm birth rate, and family history of neuropsychiatric disorders between gene-positive/suspicious positive and gene-negative children with GDD (*p* > 0.05). The incidence of epilepsy was higher in the gene-positive/suspicious positive group compared to the gene-negative group (10.2% *vs*. 1.5%, *p* = 0.034), as was the rate of an abnormal head circumference (23.7% vs. 6.0%, *χ2* = 8.080, *p* = 0.004). However, no significant difference was found in the rate of macrocephaly between the gene-positive/suspicious positive and gene-negative groups. The rate of microcephaly (15.3% *vs*. 3.0%, *p* = 0.015) was higher in the gene-positive/suspicious positive group than the gene-negative group (Table 1).

Regarding neurodevelopmental outcomes, only 2 children achieved a score in the GDS-C practical reasoning area, while the others could not complete this assessment. Consequently, we focused on the GDS-C AQ, BQ, CQ, DQ, EQ, and GQ as indicators of developmental characteristics. The results showed that the AQ, EQ, and GQ were significantly lower in the gene-positive/suspicious positive group than the gene-negative group. However, no significant differences were observed in the BQ, CQ, and DQ between the two groups (Table 2).

**Table 2:** Comparison of GDS-C between gene-positive/suspicious positive group and gene-negative group of GDD^a^.

GDS-C	Positive/suspicious positive group(*n* = 59)	Negative group (*n* = 67)	t/u	*p*
AQ	54.31 ± 18.60	64.10 ± 14.13	−3.351	0.001**
BQ	49.51 ± 16.60	51.00 ± 15.37	−0.408	0.683
CQ	38.61 ± 19.15	39.39 ± 16.56	−0.528	0.597
DQ	50.42 ± 16.32	53.34 ± 14.44	−1.065	0.289
EQ	51.17 ± 20.64	59.06 ± 16.19	−2.400	0.018*
GQ	48.25 ± 16.61	53.35 ± 11.36	−2.032	0.044*

*AQ* the development quotient of locomotor, *BQ* the development quotient of personal–social skills, *CQ* the development quotient of hearing–speech, *DQ* the development quotient of eye–hand coordination, *EQ* the development quotient of performance, *GDD* golal developmental delay, *GDS-C* Griffiths development scales Chinese edition, *GQ* general quotient.

^a^Data are mean ± SD.

*p < 0.05; **p < 0.01.

### Comparison of the gene-positive/suspicious positive rate in children with GDD according to developmental Level

**Fig. 1:**
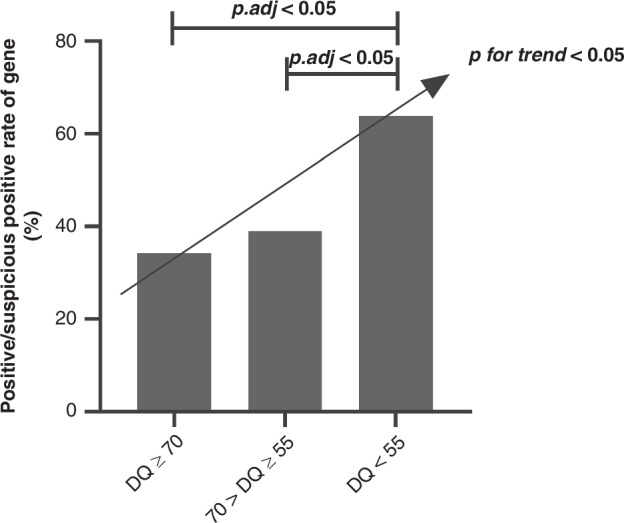
Comparison of gene-positive/suspicious positive rate in GDD with different levels of locomotor in GDS-C. *GDD* global developmental delay, *GDS-C* the chinese version of the Griffiths Mental Development Scales, *p.adj* P value corrected by Bonferroni correction, *p for trend*, the result of the chi-square trend test.

The gene-positive/suspicious positive rate varied by developmental level in the locomotor domain, 34.2% in the normal or mild defect group, 39.0% in the moderate defect group, and 63.8% in the severe defect group (*χ²* = 8.889, *p* = 0.012) (Table 3). Pairwise comparisons revealed no significant difference between the moderate defect and normal or mild defect groups (*p.adj* = 1.000), nor between the moderate defect and severe defect groups (*p.adj* = 0.062). However, a significant difference was found between the severe defect and normal or mild defect groups (*p.adj* = 0.020). The chi-squared trend test showed that the gene-positive/suspicious positive rate tended to increase with a reduction in the GDS-C AQ (*χ2* = 7.721, *p* = 0.005) (Fig. 1). No differences in the gene-positive/suspicious positive rate were observed across different levels of the GDS-C BQ, CQ, DQ, EQ, and GQ (*p* > 0.05) (Table 3).

**Table 3:** Comparison of gene-positive/suspicious positive rate in GDD with different levels of GDS-C.

	normal-mild defect (*n*/*N*, %)	moderate defect (*n*/*N*, %)	severe defect (*n*/*N*, %)	*χ2*	*p*
AQ	13/38, 34.2%	16/41, 39.0%	30/47, 63.8%	8.889	0.012*
BQ	8/17, 47.1%	17/29, 58.6%	34/80, 42.5%	2.222	0.329
CQ	5/9, 55.6%	8/14, 57.1%	46/103, 44.7%	1.068	0.586
DQ	7/16, 43.8%	15/38, 39.5%	37/72, 51.4%	1.488	0.475
EQ	11/29, 37.9%	14/35, 40.0%	34/62, 54.8%	3.175	0.204
GQ	4/9, 44.4%	20/43, 46.5%	35/74, 47.3%	0.029	0.986

*AQ* the development quotient of locomotor, *BQ* the development quotient of personal–social skills, *CQ* the development quotient of hearing–speech, *DQ* the development quotient of eye–hand coordination, *EQ* the development quotient of performance, *GDD* global developmental delay, *GDS-C* the Chinese version of the Griffiths Mental Development Scales, *GQ* general quotient, *n* number of children with positive/suspicious positive gene, *N* the total number of children in this subgroup.

*p < 0.05.

## Discussion

This study aimed to investigate the clinical features and genetic underpinnings of GDD in a cohort of 126 children through trio- WES and proband WGS. The key findings highlight the significant association between genetic variations and clinical characteristics in GDD, particularly with regard to the presence of epilepsy, microcephaly, and developmental delays across various domains. Notably, we observed that the gene-positive/suspicious positive group exhibited lower DQs in locomotor, performance, and GQ compared to the gene-negative group. Furthermore, a higher gene-positive/suspicious positive rate was associated with more severe locomotor impairments, suggesting that motor developmental delays could serve as a critical indicator for genetic testing in children with GDD.

### Gene-positive/suspicious positive rates in GDD

In this study, we found that 46.8% of the GDD cohort had genetic mutations classified as gene-positive or suspiciously positive. This rate aligns with previous studies, which reported gene-positive rates ranging from 36.07% to 66.7% in GDD populations,^4–8,15^ and is higher than the previous average positive rate of 42% for WES in GDD.^16^ The incorporation of WGS in this study allowed for the detection of structural variations and non-exonic sequence changes that would be missed by WES alone. These findings underscore the superior diagnostic capability of WGS in detecting complex genetic alterations, which can play a critical role in identifying pathogenic factors associated with neurodevelopmental disorders, particularly in cases with structural variations or non-coding mutations.^17^ This suggests that WGS could be considered a more comprehensive diagnostic tool for GDD and related intellectual disabilities.

### Clinical features associated with genetic variations

Our study revealed that children with genetically confirmed or suspicious genetic abnormalities were more likely to present with comorbid epilepsy and microcephaly, compared to those in the gene-negative group. These findings are consistent with previous research suggesting that children with GDD and ID, including those with autism spectrum disorder (ASD), often have higher rates of comorbid conditions, such as congenital heart disease, abnormal head circumference, reproductive and urinary malformations, hearing problems and epilepsy.^18–27^ Genetic abnormalities, such as those affecting the *PTEN* and *FZR1* genes have also been linked to the concurrent occurrence of developmental delay, epilepsy, and abnormal head circumference.^28,29^ Understanding the genetic mechanisms behind these associations can enhance diagnostic accuracy and inform treatment strategies for both epilepsy and other neurodevelopmental disorders.^30^

### Developmental quotients and genetic findings

While prior studies have indicated a higher likelihood of genetic abnormalities in children with severe GDD/ID,^31,32^ few have provided a detailed comparison of developmental outcomes between gene-positive and gene-negative children. Our results show that, within the GDD cohort, the gene-positive/suspicious positive group exhibited lower developmental quotients in the non-verbal domains (locomotor, performance, and general quotients) compared to the gene-negative group. Interestingly, no significant differences were found in the language or personal–social quotients between the two groups. This suggests that genetic factors may have a more pronounced impact on certain developmental domains, such as motor skills and overall performance, rather than language or social development.

Furthermore, the rate of gene positivity varied significantly across different developmental levels, particularly within the locomotor domain. Children with poorer locomotor ability were more likely to have a positive or suspicious genetic test result. This finding is particularly relevant, as previous studies on ASD have suggested that locomotor delays, while not a core diagnostic feature, may serve as an early clinical indicator of developmental disorders.^33^ In ASD, locomotor ability has been shown to be a more sensitive indicator of the severity genetic mutations compared to cognitive abilities such as the intelligence quotient.^34^ Our study corroborates this finding, suggesting that locomotor impairments may serve as a valuable indicator for initiating genetic testing in children with GDD. However, the sample size of this study is relatively small, so future studies with larger cohorts is necessary to validate these findings. Motor and cognitive abilities are frequently posited to be intricately interconnected. Empirical evidence has demonstrated that locomotor skills directly enhance visuomotor integration and, by extension, indirectly improve mathematical abilities in typically developing preschool children.^35^ Furthermore, locomotor impairments have been significantly associated with diminished social communication capabilities in children with ASD,^36^ with locomotor performance serving as a predictive indicator for both social communication skills and the severity of repetitive behaviors in ASD.^37^ Although children with GDD/ID commonly present with delayed locomotor development, there remains a notable paucity of research investigating the relationship between locomotor skills and cognitive function in this population. This represents a critical gap in the literature that warrants further exploration.

### Strengths and limitations

This study offers several strengths, including its focus on the genetic and developmental profiles of children with GDD, as well as its examination of how genetic status correlates with clinical outcomes. Despite these strengths, the study has several limitations. First, the relatively small sample size limits the generalizability of our findings, which may not represent the broader population of children with GDD. Future research should aim to expand the sample size and incorporate diverse regions. Second, our study’s cross-sectional design restricts our ability to examine the long term prognosis of children with different clinical phenotypes and genetic background. Longitudinal studies that follow children over time would provide more comprehensive insights into the developmental trajectories of genetically positive and negative children with GDD.

## Conclusion

In conclusion, children with GDD and genetic abnormalities exhibited poorer locomotor, performance, and general developmental quotients compared to those without genetic mutations. These findings emphasize the importance of genetic testing in identifying developmental impairments and guiding clinical decision-making in GDD management.

## Supplementary information

Supplemental TABLE S1

Supplementary TABLE S2

## Data Availability

All the data and materials are available. The datasets used and analyzed during the current study are available from the corresponding author upon reasonable request.
