# Supplementary material for: Global developmental delay: comparison of developmental profiles between gene-positive/suspicious positive and gene-negative cases
Source: Pediatr Res. 2025 Apr 30;98(5):1795–800. doi: 10.1038/s41390-025-04085-y (PMC12602308; doi:10.1038/s41390-025-04085-y)
Supplement: Supplementary file 2 — Supplementary TABLE S2 [file 41390_2025_4085_MOESM2_ESM.pdf]

Supplemental TABLE S2. Summary of pathogenic or likely pathogenic CNVs of GDD

Number	Gender	CNV type	Chromosomal locations	CNV size	Known syndromes	Or	N/R
44	male	duplication	chr15:22646194-32734856	10.09 Mb	/	d	N
5	female	duplication	chr6:162622162- 162622284	123 bp	/	m	N
45	male	deletion	chrX:31225175-31498184	273.01 Kb	DMD/BMD	m	N
46	female	deletion	chr8:116085-7220468	7.10 Mb	/	d	N
47	male	deletion	chr1:153781634-153846579	64.95 Kb	/	d	N
48	female	deletion	chr22:50654145-51220722	566.58 Kb	Phelan-Mcdermid syndrome	d	N
49	female	deletion	chr22:46108868-51304566	5.20 Mb	Phelan-Mcdermid syndrome	d	N
50	male	deletion	chr9:73992310-73997780	5.47Kb	/	d	N
51	female	deletion	chrX:154131556-154539039	407.48 Kb	/	d	N
52	male	deletion	chrX:29002994-29009596	6.60 Kb	/	d	N
53	male	duplication	chr16:2967504 9-30199570	524 .52Kb	16P11.2 microduplication syndrome	m	N
54	female	duplication	chr1:199996975-211192598	11.52 Mb	/	d	N
55	male	deletion	chrX:32690592- 32930592	240 Kb	DMD/BMD	m	N
56	female	deletion	chr22:49748364 -51244566	1.50 Mb	Phelan-Mcdermid syndrome	d	N
57	male	duplication	chr12:57486325- 57606670	120.35 Kb	/	d	N
58	female	deletion	chr11:70068674 -70334 277	265.60 Kb	/	d	N
59	male	deletion	chr15:22747540- 23264134	516.60 Kb	/	d	N

CNV, copy number variation; d, de novo; GDD, global developmental delay; m, maternal; N, novel; Or, origin; p, paternal; R, reporte
